# The Relative Impact of Urinary and Sexual Function vs Bother on Health Utility for Men With Prostate Cancer

**DOI:** 10.1093/jncics/pkaa044

**Published:** 2020-05-25

**Authors:** Chang Wook Jeong, Annika Herlemann, Janet E Cowan, Jeanette M Broering, Renske M T ten Ham, Leslie S Wilson, Peter R Carroll, Matthew R Cooperberg

**Affiliations:** p1 Diller Family Comprehensive Cancer Center, Department of Urology, University of California, San Francisco, CA, USA; p2 Department of Urology, Seoul National University Hospital, Seoul National University College of Medicine, Seoul, Republic of Korea; p3 Department of Urology, Ludwig-Maximilians-University of Munich, Munich, Germany; p4 Health Policy and Economics, Department of Clinical Pharmacy, University of California, San Francisco, CA, USA; p5 Division of Pharmacoepidemiology and Clinical Pharmacology, Utrecht Institute for Pharmaceutical Sciences, Utrecht University, Utrecht, the Netherlands; p6 Department of Epidemiology and Biostatistics, University of California, San Francisco, CA, USA

## Abstract

Function and bother are related but distinct aspects of health-related quality of life. The objective of this study was to compare quantitatively the relative impacts of function and bother in urinary, sexual, and bowel outcomes on health utility as a reflection of health-related quality of life in men with prostate cancer. Our analysis included participants in the Cancer of the Prostate Strategic Urologic Research Endeavor utility supplementary study, with a final cohort of 1617 men. Linear regression on the patients’ function and bother summary scores (0-100) from the University of California, Los Angeles Prostate Cancer Index was performed to predict bias-corrected health utilities. Urinary and sexual bother were associated with each health utility, and their coefficients were 3.7 and 20.8 times greater, respectively, than those of the corresponding function. To our knowledge, our study provides the first quantitative and direct comparison of the impacts of function vs bother on health utility.

Health-related quality of life (HRQoL) is a critical determinant of satisfactory outcomes after prostate cancer (PCa) management and is affected differently by various treatments (1). Besides function, bother is an indication of how much the symptom interferes with the patient’s activities or how much the symptom annoys the patient (2). Function and bother are weakly correlated, and they may weigh differently on a patient’s ultimate subjective HRQoL (3–9). Therefore, for domains including urinary, sexual, and bowel outcomes, both function and bother should be measured and evaluated separately for men with PCa (2).

Standardized patient-reported HRQoL questionnaires, such as the University of California, Los Angeles Prostate Cancer Index (UCLA-PCI), measure function and bother separately for each HRQoL domain. However, the extent to which the subscore for function or bother reflects subjective HRQoL remains unclear.

The HRQoL score is needed to calculate a health utility based on prederived weights or formulae (10,11). Health utilities are preference-based measures for particular health states made under conditions of uncertainty (10). They quantify this final perception by using standardized values ranging from 0 (death) to 1 (perfect health) and are used to calculate quality-adjusted life-years (12). For example, if a man with metastatic PCa and a utility value of 0.83 (13) lives 10 years, his corresponding quality-adjusted life-years are 8.3.

Recently, our group determined robust utilities for various outcomes among men participating in the Cancer of the Prostate Strategic Urologic Research Endeavor (CaPSURE) (13). In this follow-up study, we aimed to assess quantitatively the impact of function vs bother on utilities in 3 domains as a reflection of HRQoL among men with PCa.

We used data from 1617 patients in the CaPSURE utility supplementary study (CaPSURE-USS), which was a nested cross-sectional survey that measured utilities using the standard gamble method (13). The original study (CaPSURE) was approved (University of California, San Francisco IRB #10-00881), and all participants signed an informed consent form. To extend to CaPSURE-USS, we modified the study protocol (modification #0379720), and this modification was approved on January 4, 2012. Thus, the CaPSURE-USS was conducted under the same informed consent form signed at enrollment into original CaPSURE study. We separately measured utility values for 5 domains (urinary, sexual, bowel, PCa, and overall health) based on each patient’s condition. We adapted a validated paper instrument for PCa (14). A full copy of the instruments and detailed methodology can be found in our previous publication (13). Patients’ characteristics are summarized in Table 1.

**Table 1.** Basic characteristics of the patients (N = 1617)^a^

Variable	No. (%)
Age at diagnosis, mean ± SD (median, IQR), y	63.5 ± 7.7 (63.0, 58.0-69.0)
Age at survey, mean ± SD (median, IQR), y	72.8 ± 8.2 (73.0, 67.0-79.0)
Duration since diagnosis, mean ± SD (median, IQR), y	8.8 ± 4.0 (9.0, 6.0-11.0)
Race	
White	1511 (93.4)
Black	68 (4.2)
Latino	12 (0.7)
Other	26 (1.6)
Comorbidity	
0	283 (17.5)
1-2	842 (52.1)
≥3	359 (22.2)
Unknown	133 (8.2)
PSA at diagnosis, mean ± SD (median, IQR), ng/mL	7.6 ± 10.5 (5.6, 4.3-7.8)
ISUP grade group	
1	1071 (66.2)
2	276 (17.1)
3	122 (7.5)
4	70 (4.3)
5	37 (2.3)
Unknown	41 (2.5)
Clinical T stage at diagnosis	
T1	867 (53.6)
T2	636 (39.3)
T3	26 (1.6)
TX	88 (5.4)
Clinical N stage at diagnosis	
N0	341 (21.1)
N1	6 (0.4)
Nx	1270 (78.5)
Clinical M stage at diagnosis	
M0	529 (32.7)
M1	6 (0.4)
Mx	1082 (66.9)
Primary treatment	
Active surveillance or watchful waiting	74 (4.5)
Radical prostatectomy	1041 (64.4)
Brachytherapy	168 (10.4)
External beam radiation therapy	133 (8.2)
Cryotherapy	62 (3.8)
Androgen deprivation therapy	88 (5.4)
Others or unknown	51 (3.2)
Disease status at survey	
Active surveillance or watchful waiting without treatment	54 (3.3)
No evidence of disease	1144 (70.7)
Biochemical recurrence	77 (4.8)
Remission	248 (15.3)
Androgen deprivation therapy without metastasis	22 (1.4)
Metastasis	24 (1.5)
Unknown	48 (3.0)
UCLA-PCI at the survey, mean ± SD (median, IQR)	
Urinary function	77.6 ± 22.9 (81.7, 65.0-100)
Urinary bother	78.1 ± 25.5 (75.0, 75.0-100)
Bowel function	87.7 ± 14.0 (93.8, 82.5-100)
Bowel bother	86.7 ± 21.8 (100.0, 75.0-100)
Sexual function	28.1 ± 27.3 (18.8, 4.2-47.6)
Sexual bother	46.3 ± 39.1 (50.0, 0-75.0)
Health utility, mean ± SD (median, IQR)	
Urinary function	0.915 ± 0.123 (0.971, 0.890-0.973)
Bowel function	0.919 ± 0.120 (0.967, 0.933-0.967)
Sexual function	0.877 ± 0.154 (0.963, 0.815-0.975)
Prostate cancer health	0.866 ± 0.154 (0.932, 0.774-0.973)
Overall health	0.892 ± 0.144 (0.968, 0.877-0.973)

a IQR = interquartile range; ISUP = International Society of Urological Pathologists; PSA = prostate specific antigen; UCLA-PCI = University of California, Los Angeles Prostate Cancer Index.

Patients’ function and bother were assessed using the UCLA-PCI standardized scores (0-100, with higher numbers indicating better function or less bother). Then the summarized score for each domain was calculated as an average value of all items in that domain. The primary outcome was the utility for each domain (urinary, sexual, and bowel) status. We used bias-corrected utilities using 1-parameter weighting (13,15,16). The detailed methodology is in the Supplementary Methods (available online).

We used linear regressions to predict the utility associated with the summarized function and bother scores in each domain as simple models (function and bother, 2 predictors). Then we generated linear regression models using the scores of all individual UCLA-PCI questions to investigate detailed associations in a full model (all questions as predictors).

To match scales between the standardized UCLA-PCI scores (0-100) and utilities (0-1), we divided the UCLA-PCI scores by 100. We used adjusted *R^2^*, *F* statistics, root-mean-square error, and mean absolute error to evaluate the goodness of fit of models. The importance of individual variables was calculated as the relative contribution to variance explained (RCVE) (17). The formula is as follows: (*R^2^*_full_ − *R^2^*_reduced_)/ *R^2^*_full_, where *R^2^*_full_ represents the share of explained variability by all the predictors in the model, whereas *R^2^*_reduced_ represents the explained variability without the specific variable whose contribution is being evaluated. We performed subgroup analyses comparing the utilities predicted by the simple models with the actual utility values according to the initial treatments, disease status at the survey, and each functional status, respectively.

In the regression models, urinary and sexual bother demonstrated a statistically significant association with each utility, whereas the corresponding summarized function scores were not associated (Table 2). The simple model coefficients for urinary and sexual bother were 3.7 and 20.8 times greater than those of each function, respectively. However, the utility for the bowel domain showed a statistically significant and higher association with the function compared with bother (ratio of bother to function = 0.4). These trends were similar in both the full and reduced models.

**Table 2.** Linear regression models^a^

Predictors	Simple model			Full model		
	Coef.	SE	*P* ^b^	Coef.	SE	*P* ^b^
Utility for urinary health						
Urinary bother	0.074	0.019	<.001	0.069	0.021	.001
Urinary function	0.020	0.021	.35	—	—	—
Question 1 (urine leak)	—	—	—	−0.015	0.014	.29
Question 2 (urinary control)	—	—	—	0.058	0.028	.04
Question 3 (diapers per day)	—	—	—	0.024	0.022	.91
Question 4 (dripping or wetting)	—	—	—	−0.015	0.025	.55
Question 5 (leakage interfering with sexual activity)	—	—	—	0.003	0.013	.84
Utility for sexual health						
Sexual bother	0.083	0.012	<.001	0.078	0.013	<.001
Sexual function	0.004	0.016	.82	—	—	—
Question 1 (level of sexual desire)	—	—	—	−0.001	0.016	.96
Question 2 (ability to have an erection)	—	—	—	−0.006	0.032	.86
Question 3 (ability to reach orgasm)	—	—	—	0.012	0.019	.52
Question 4 (quality of erection)	—	—	—	−0.042	0.020	.04
Question 5 (frequency of erection)	—	—	—	0.017	0.026	.51
Question 6 (morning or nocturnal erection)	—	—	—	0.006	0.024	.79
Question 7 (achieving intercourse)	—	—	—	0.052	0.030	.08
Question 8 (ability of sexual function)	—	—	—	−0.003	0.029	.91
Utility for bowel health						
Bowel bother	0.041	0.022	.06	0.028	0.025	.25
Bowel function	0.109	0.035	.002	—	—	—
Question 1 (rectal urgency)	—	—	—	0.026	0.017	.11
Question 2 (loose or liquid stool)	—	—	—	0.032	0.018	.08
Question 3 (distress due to bowel movements)	—	—	—	0.035	0.026	.17
Question 4 (crampy pain in abdomen or pelvis)	—	—	—	−0.046	0.017	.006

a Coef. = coefficient; SE = standard error.

b *P* values of *t* statistics (2-sided).

With regard to urinary function, subjective urinary control (question 2 in the urinary domain) was found to have the highest association with urinary utility. Achieving intercourse (question 7 in the sexual domain) was most closely associated with sexual utility. All functional questions in the bowel domain (with the exception of question 4, which concerned crampy pain) were correlated with bowel utility.

All results for goodness of fit are shown in Supplementary Table 1 (available online). Supplementary Table 2 (available online) presents the RCVE of all linear models. The RCVE of urinary and sexual bother were 8.7 and 82 times greater than those of each function in simple models, respectively. Subgroup analyses demonstrated a fair correlation between the estimated utilities using linear regression models and the actual utilities, especially when the patient number is large (Supplementary Tables 3 and 4, available online).

To our knowledge, our study is the first quantitative and direct comparison evaluating the relative impact of function vs bother outcomes on health utilities among men with PCa. We found that bother had a greater impact on urinary and, in particular, sexual HRQoL compared with function. This result is not entirely unexpected, because bother reflects how a patient perceives a given level of function. The high proportion of elderly men with baseline erectile dysfunction and decreased sexual activity may contribute to the weak correlation between sexual bother and function and their impacts on this health utility. Older men may also adapt more easily over time to sexual dysfunction than to urinary incontinence or bowel dysfunction (10). Because bother associates more tightly with utility than function, we do stress that bother should be measured in addition to function for any HRQoL studies, because bother may also reflect nonclinical factors such as patient preferences and expectations.

The results regarding the bowel domain should be interpreted cautiously, because only 3.5% of men suffered from bowel issues. All results of goodness of fit were not high and low variance explained can be limitations of the models. Other limitations of our study include the restricted generalizability of our results because of the characteristics of the study population: 93.4% were white men living in the United States, and HRQoL may vary by race or ethnicity and/or geographic region. The majority of men in CaPSURE-USS were long-term survivors of PCa and relatively old at the time of the survey (mean age of 73 years). The findings should, therefore, be applied cautiously to men with short-term follow-up or to younger patients. The UCLA-PCI focuses on urinary incontinence rather than irritative or obstructive symptoms, so the results may not extend to these other subdomains and are more relevant to surgical than to radiation patients.

Despite these limitations, our study helps us understand how HRQoL is influenced by patients’ functional status and recovery process. Our results suggest that urinary and sexual bother have greater impact on HRQoL than function per se in men with PCa.

## Funding

CaPSURE was funded until 2007 by TAP Pharmaceutical Products Inc. It is currently funded by US Department of Defense grant W81XWH-13-2-0074 and the University of California, San Francisco, Department of Urology. This study was also partly supported by a grant from the National R&D Program for Cancer Control, Ministry of Health and Welfare, Republic of Korea (HA17C0039).

## Notes

**Role of the funder:** None of the sponsors had any access to the data or any influence on or access to the analysis plan, the results, or the manuscript.

Disclosures: All authors report no conflict of interest.

## Supplementary Material

pkaa044_Supplementary_Data
