# Supplementary material for: The Relative Impact of Urinary and Sexual Function vs Bother on Health Utility for Men With Prostate Cancer
Source: JNCI Cancer Spectr. 2020 May 25;4(5):pkaa044. doi: 10.1093/jncics/pkaa044 (PMC7583149; doi:10.1093/jncics/pkaa044)
Supplement: pkaa044_Supplementary_Data [file pkaa044_supplementary_data.docx]

**Supplementary online content**

**Supplementary material and method**

The Cancer of the Prostate Strategic Urologic Research Endeavor (CaPSURE) is a national disease registry that prospectively enrolls men with PCa from 45 predominantly community-based urology practices in the United States since 1995 [1, 2]. The CaPSURE utility supplementary study (CaPSURE-USS) was a nested cross-sectional survey that measured utilities using the standard gamble (SG) method, generally considered to be the most reliable approach [3]. We also collected eight other related questionnaires including the University of California, Los Angeles Prostate Cancer Index (UCLA-PCI) along with paper SG instrument.

Among 1,740 men who completed the CaPSURE-USS, 1,617 comprised the final analytic cohort, after excluding 123 with missing or incomplete data on urinary, sexual, or bowel outcomes. Patients’ basic characteristics are presented in Table 1 of the main article.

The primary outcome was the SG utility value for each domain (urinary/sexual/bowel) status. Because crude SG utility value is upwardly biased [4-7], we used bias-corrected utilities using a one-parameter correction function [6, 7]. For this calculation, we applied the probability weighting parameter γ = 0.61 following the most common assumption [3, 7].

Patients’ function and bother were assessed using the UCLA-PCI standardized scores (0-100, with higher numbers indicating better function/less bother) at the time of CaPSURE-USS. UCLA-PCI consists of five urinary function, eight sexual function, and four bowel function items, and one bother question for each domain [8]. Standardized score for each item was assigned from 0-100 following the instruction. Then summarized score for each domain was calculated as an average value of all items in that domain.

We used linear regression to correlate the utility associated with the summarized function and bother scores in each domain as *simple* models. We estimated and compared the impact on HRQoL using regression model coefficients. The purpose of the regression modeling was for comparing coefficients between function and bother score, not for better prediction, thus we did not use interaction term adjusting for multicollinearity. Then, we generated linear regression models using the standardized scores of all 20 individual questions to investigate detailed associations in a *full* model. At this step, we can find significantly associated items (aspects of HRQoL) to utility values.

To match scales between the standardized UCLA-PCI scores (0-100) and utilities (0-1), we divided the scores by 100 in the regression model. We employed adjusted R-squared, F-statistics, root-mean square error (RMSE), and mean absolute error (MAE) to evaluate the goodness-of-fit of models.

To evaluate relative importance of predictors, we computed the relative contribution to variance explained (RCVE) for each linear regression model [9, 10]. RCVE compares the regression sum of squares of the final model to that of the reduced model (i.e., the final model excluding terms related to the variable of interest), to quantify the improved reduction in error of the regression model (i.e., ‘‘increased fit’’) attained by inclusion of the variable of interest.

To test the performance of the *simple* models, we performed subgroup analyses comparing the utilities predicted by the models with the actual utility values according to the initial treatments, disease status at the survey, and each functional status.

**Supplementary Table 1.** Goodness-of-fit measures for the linear regression models *

|  | Simple model | Full model |
| --- | --- | --- |
| Utility for urinary health |  |  |
| Adjusted R^2^ | 0.032 | 0.028 |
| p-value of F-statistic | <0.001 | <0.001 |
| RMSE | 0.119 | 0.119 |
| MAE | 0.116 | 0.116 |
| Utility for sexual health |  |  |
| Adjusted R^2^ | 0.043 | 0.038 |
| p-value of F-statistic | <0.001 | <0.001 |
| RMSE | 0.151 | 0.149 |
| MAE | 0.116 | 0.116 |
| Utility for bowel health |  |  |
| Adjusted R^2^ | 0.034 | 0.033 |
| p-value of F-statistic | <0.001 | <0.001 |
| RMSE | 0.117 | 0.116 |
| MAE | 0.079 | 0.078 |

* MAE = mean absolute error; RMSE = root mean squares error

**Supplementary Table 2.** Relative contribution to variance explained in each model

| Predictors | Simple model | Full model |
| --- | --- | --- |
| Utility for urinary health |  |  |
| Urinary bother | 0.26 | 0.18 |
| Urinary function | 0.03 |  |
| Question 1 (Urine leak) |  | 0.02 |
| Question 2 (Urinary control) |  | 0.09 |
| Question 3 (Diapers per day) |  | -0.10 |
| Question 4 (Dripping or wetting) |  | 0 |
| Question 5 (Leakage interfering with sexual activity) |  | -0.05 |
| Utility for sexual health |  |  |
| Sexual bother | 0.82 | 0.65 |
| Sexual function | 0.01 |  |
| Question 1 (Level of sexual desire) |  | 0 |
| Question 2 (Ability to have an erection) |  | 0 |
| Question 3 (Ability to reach orgasm) |  | 0.02 |
| Question 4 (Quality of erection) |  | 0.06 |
| Question 5 (Frequency of erection) |  | 0 |
| Question 6 (Morning or nocturnal erection) |  | 0 |
| Question 7 (Achieving intercourse) |  | 0 |
| Question 8 (Ability of sexual function) |  | 0 |
| Utility for bowel health |  |  |
| Bowel bother | 0.06 | 0.02 |
| Bowel function | 0.18 |  |
| Question 1 (Rectal urgency) |  | 0.05 |
| Question 2 (Loose or liquid stool) |  | 0.06 |
| Question 3 (Distress due to bowel movements) |  | 0.03 |
| Question 4 (Crampy pain in abdomen or pelvis) |  | 0.02 |

**Supplementary Table 3.** Subgroup sensitivity analysis by initial treatment and disease status at the survey (Predicted by simple model) *

|  | N | | Urinary health | | Sexual health | | Bowel health | |
| --- | --- | --- | --- | --- | --- | --- | --- | --- |
|  |  |  | Actual utilities | Predicted utilities | Actual utilities | Predicted utilities | Actual utilities | Predicted utilities |
| Initial Treatment | | | |  |  |  |  |  |
| AS | | 62 | 0.887 ± 0.158 | 0.919 ± 0.022 | 0.844 ± 0.193 | 0.875 ± 0.033 | 0.900 ± 0.144 | 0.912 ± 0.026 |
| WW | | 12 | 0.892 ± 0.151 | 0.917 ± 0.025 | 0.842 ± 0.173 | 0.873 ± 0.037 | 0.867 ± 0.157 | 0.916 ± 0.019 |
| Radical prostatectomy | | 1041 | 0.920 ± 0.118 | 0.914 ± 0.023 | 0.877 ± 0.152 | 0.877 ± 0.032 | 0.927 ± 0.111 | 0.921 ± 0.021 |
| Brachytherapy | | 168 | 0.909 ± 0.129 | 0.922 ± 0.019 | 0.884 ± 0.140 | 0.880 ± 0.033 | 0.915 ± 0.110 | 0.918 ± 0.023 |
| EBRT | | 133 | 0.906 ± 0.139 | 0.914 ± 0.024 | 0.874 ± 0.171 | 0.871 ± 0.035 | 0.909 ± 0.134 | 0.914 ± 0.026 |
| Cryotherapy | | 62 | 0.906 ± 0.132 | 0.915 ± 0.023 | 0.891 ± 0.142 | 0.864 ± 0.031 | 0.888 ± 0.143 | 0.918 ± 0.026 |
| ADT | | 88 | 0.907 ± 0.119 | 0.917 ± 0.022 | 0.882 ± 0.157 | 0.872 ± 0.036 | 0.887 ± 0.155 | 0.912 ± 0.029 |
| Other | | 51 | 0.928 ± 0.098 | 0.916 ± 0.021 | 0.879 ± 0.151 | 0.869 ± 0.033 | 0.919 ± 0.133 | 0.915 ± 0.025 |
| Disease status at survey | | | |  |  |  |  |  |
| AS without Tx | | 44 | 0.902 ± 0.154 | 0.922 ± 0.019 | 0.859 ± 0.196 | 0.874 ± 0.033 | 0.925 ± 0.109 | 0.914 ± 0.023 |
| WW without Tx | | 10 | 0.890 ± 0.170 | 0.920 ± 0.021 | 0.830 ± 0.193 | 0.867 ± 0.038 | 0.863 ± 0.172 | 0.913 ± 0.020 |
| NED | | 1144 | 0.918 ± 0.120 | 0.917 ± 0.022 | 0.879 ± 0.149 | 0.877 ± 0.033 | 0.922 ± 0.114 | 0.922 ± 0.020 |
| BCR | | 77 | 0.882 ± 0.168 | 0.906 ± 0.026 | 0.852 ± 0.177 | 0.872 ± 0.033 | 0.899 ± 0.147 | 0.914 ± 0.028 |
| Remission | | 248 | 0.915 ± 0.117 | 0.912 ± 0.025 | 0.885 ± 0.155 | 0.873 ± 0.034 | 0.909 ± 0.134 | 0.914 ± 0.028 |
| ADT without Mets | | 22 | 0.885 ± 0.143 | 0.909 ± 0.025 | 0.838 ± 0.202 | 0.863 ± 0.031 | 0.926 ± 0.143 | 0.907 ± 0.027 |
| Metastasis | | 24 | 0.914 ± 0.099 | 0.918 ± 0.014 | 0.860 ± 0.143 | 0.871 ± 0.036 | 0.932 ± 0.082 | 0.908 ± 0.029 |

The data are presented as mean ± standard deviation.

* ADT = androgen deprivation therapy; AS = active surveillance; BCR = biochemical recurrence; EBRT = external beam radiation therapy; Mets = metastasis; NED = no evidence of disease; Tx = treatment; WW = watchful waiting

**Supplementary Table 4.** Subgroup sensitivity analysis by functional status (Predicted by simple model) *

|  | | N | Actual utilities  Mean ± SD | Predicted utilities,  Mean ± SD |
| --- | --- | --- | --- | --- |
| Urinary health | | |  |  |
| Continence | | |  |  |
| No pad/day | 1298 | | 0.922 ± 0.116 | 0.922 ± 0.017 |
| ≥ 1 pad/day | 303 | | 0.886 ± 0.141 | 0.889 ± 0.024 |
| IPSS total score | | |  |  |
| Mild | 984 | | 0.931 ± 0.105 | 0.922 ± 0.018 |
| Moderate | 524 | | 0.896 ± 0.140 | 0.907 ± 0.023 |
| Severe | 70 | | 0.847 ± 0.158 | 0.884 ± 0.025 |
| IPSS QoL score | | |  |  |
| 0 (best) | 242 | | 0.941 ± 0.105 | 0.932 ± 0.008 |
| 1 | 459 | | 0.933 ± 0.102 | 0.925 ± 0.013 |
| 2 | 489 | | 0.920 ± 0.115 | 0.914 ± 0.019 |
| 3 | 224 | | 0.876 ± 0.150 | 0.901 ± 0.024 |
| 4 | 100 | | 0.874 ± 0.146 | 0.894 ± 0.023 |
| 5 | 50 | | 0.872 ± 0.130 | 0.879 ± 0.026 |
| 6 (worst) | 9 | | 0.788 ± 0.248 | 0.847 ± 0.009 |
| Sexual health | | |  |  |
| Potency |  | |  |  |
| Potent | 319 | | 0.901 ± 0.132 | 0.899 ± 0.023 |
| Impotent | 1233 | | 0.870 ± 0.159 | 0.869 ± 0.032 |
| SHIM score |  | |  |  |
| No ED | 160 | | 0.917 ± 0.114 | 0.910 ± 0.020 |
| Mild | 164 | | 0.877 ± 0.142 | 0.888 ± 0.022 |
| Mild to mod. | 100 | | 0.866 ± 0.164 | 0.875 ± 0.024 |
| Moderate | 57 | | 0.893 ± 0.148 | 0.875 ± 0.025 |
| Severe | 1077 | | 0.870 ± 0.160 | 0.868 ± 0.033 |
| Bowel health | | |  |  |
| Bowel problem | | |  |  |
| No | 1519 | | 0.923 ± 0.114 | 0.921 ± 0.020 |
| Yes | 56 | | 0.822 ± 0.195 | 0.866 ± 0.031 |

* ED = erectile dysfunction; IPSS = International Prostate Symptom Score; SHIM = Sexual Health Inventory for Men; QoL = quality of life

**References**

1. Cooperberg MR, Carroll PR. Trends in Management for Patients With Localized Prostate Cancer, 1990-2013. JAMA 2015;314(1):80-2.

2. Lubeck DP, Litwin MS, Henning JM, et al. The CaPSURE database: a methodology for clinical practice and research in prostate cancer. CaPSURE Research Panel. Cancer of the Prostate Strategic Urologic Research Endeavor. Urology 1996;48(5):773-7.

3. Jeong CW, Cowan JE, Broering JM, et al. Robust health utility assessment among long-term survivors of prostate cancer: Results from the Cancer of the Prostate Strategic Urologic Research Endeavor Registry. Eur Urol 2019;76(6):743-51.

4. Bleichrodt H, Pinto JL, Wakker PP. Making Descriptive Use of Prospect Theory to Improve the Prescriptive Use of Expected Utility. Management Science 2001;47(11):1498-1514.

5. Doctor JN, Bleichrodt H, Lin HJ. Health utility bias: a systematic review and meta-analytic evaluation. Med Decis Making 2010;30(1):58-67.

6. Tversky A, Kahneman D. Advances in Prospect Theory: Cumulative Representation of Uncertainty. J Risk Uncertain 1992;5:297-323.

7. van Osch SM, Wakker PP, van den Hout WB, et al. Correcting biases in standard gamble and time tradeoff utilities. Med Decis Making 2004;24(5):511-7.

8. Litwin MS, Hays RD, Fink A, et al. The UCLA Prostate Cancer Index: development, reliability, and validity of a health-related quality of life measure. Med Care 1998;36(7):1002-12.

9. [Kelkar YD](https://www.ncbi.nlm.nih.gov/pubmed/?term=Kelkar%20YD%5BAuthor%5D&cauthor=true&cauthor_uid=18032720), [Tyekucheva S](https://www.ncbi.nlm.nih.gov/pubmed/?term=Tyekucheva%20S%5BAuthor%5D&cauthor=true&cauthor_uid=18032720), [Chiaromonte F](https://www.ncbi.nlm.nih.gov/pubmed/?term=Chiaromonte%20F%5BAuthor%5D&cauthor=true&cauthor_uid=18032720), [Makova KD](https://www.ncbi.nlm.nih.gov/pubmed/?term=Makova%20KD%5BAuthor%5D&cauthor=true&cauthor_uid=18032720). The genome-wide determinants of human and chimpanzee microsatellite evolution. [Genome Res.](https://www.ncbi.nlm.nih.gov/pubmed/?term=The+genome-wide+determinants+of+human+and+chimpanzee+microsatellite+evolution) 2008;18(1):30-8.

10. [Campos-Sánchez R](https://www.ncbi.nlm.nih.gov/pubmed/?term=Campos-S%C3%A1nchez%20R%5BAuthor%5D&cauthor=true&cauthor_uid=24809961), [Kapusta A](https://www.ncbi.nlm.nih.gov/pubmed/?term=Kapusta%20A%5BAuthor%5D&cauthor=true&cauthor_uid=24809961), [Feschotte C](https://www.ncbi.nlm.nih.gov/pubmed/?term=Feschotte%20C%5BAuthor%5D&cauthor=true&cauthor_uid=24809961), [Chiaromonte F](https://www.ncbi.nlm.nih.gov/pubmed/?term=Chiaromonte%20F%5BAuthor%5D&cauthor=true&cauthor_uid=24809961), [Makova KD](https://www.ncbi.nlm.nih.gov/pubmed/?term=Makova%20KD%5BAuthor%5D&cauthor=true&cauthor_uid=24809961). Genomic landscape of human, bat, and ex vivo DNA transposon integrations. [Mol Biol Evol.](https://www.ncbi.nlm.nih.gov/pubmed/?term=Genomic+Landscape+of+Human%2C+Bat%2C+and+Ex+Vivo+DNA+Transposon+Integrations) 2014 Jul;31(7):1816-32.
